# Antidepressant use in the Canary Islands (Spain): a retrospective study of provincial, island and municipal trends and associated factors

**DOI:** 10.3389/fphar.2025.1511936

**Published:** 2025-07-02

**Authors:** Alexis Oliva, Vanessa Moreno, Mariana Sapino, Sandra Dévora, Susana Abdala-Kuri

**Affiliations:** ^1^ Departamento de Ingeniería Química y Tecnología Farmacéutica, Facultad de Farmacia, Universidad de La Laguna, Tenerife, Spain; ^2^ Departamento de Medicina Física y Farmacología, Facultad de Farmacia, Universidad de La Laguna, Tenerife, Spain

**Keywords:** antidepressant, depression, urban/rural, defined daily dose, observational retrospective study, pharmacoepidemiology, Canary islands, Spain

## Abstract

**Introduction:**

The presence of a diagnosis by a general practitioner is a major reason for the use of antidepressant (ADs). However, the simultaneous analysis of several and interrelated socioeconomic and demographic factors could provide a picture of the distribution of AD use in a given population across diverse geographical regions and socioeconomic backgrounds. The aim of the present study was to provide a picture of the trends in the consumption of ADs at the provincial, island and municipal levels for the period 2016–2021 in the Canary Islands (Spain), as an example of a geographically isolated area. To this end, several factors were analyzed, such as living in a rural or urban area, the population over 65 years of age, the population density or the socioeconomic status.

**Methods:**

Data were extracted from the community pharmacy wholesaler at the population level. A model with two nested fixed factors and a co-variable were used to analyze the trends in the use of ADs and the factors associated. Dispensation ADs use was measured as defined daily doses (DDD) per 1000 inhabitant per day. This provided total overall dispensation of AD and its rate of change for each island, as well as differences in dispensation at the island and municipal level.

**Results:**

Over the study period, prescription rates increased steadily at all population levels, although the level of dispensing and the rate of variation varied between island and municipalities but no between provinces. The data on the use of ADs at the level of the province and islands are more accurate because they include the entire population that is resident in that zone. At the municipal level, there is a bias, but it is difficult to quantify. The prescription patterns at municipal level were similar to those observed at island level, although with small variations in the dispensation level. Selective serotonin reuptake inhibitors (SSRIs) were the most commonly used, followed by “other ADs,” whereas the use of tricyclic ADs remained stable. 1n addition, all AD drugs are equally available regardless of the geographical area of residence or socio-economic status although the type of AD and ranking varied slightly between islands due to the differences in general medical practice.

**Conclusion:**

The observed differences in ADs use between rural and urban areas cannot be attributed to the factors of urbanization, population age, population density and socioeconomic status. However, the medical practices, the social and cultural traditions of each island, may provide insights into the underlying reasons for this variation.

## 1 Introduction

Major depressive disorder is one of the most common, burdensome and costly mental disorders in adults worldwide, affecting over 330 million people, and is now the leading cause of disability in contemporary society according to the WHO report (Arias et al., 2022).

There are pharmacological and non-pharmacological treatments, but due to lack of resources, antidepressants are more commonly used than psychological interventions. These agents should be prescribed on the basis of the best available evidence (Cipriani et al., 2018). Antidepressants (ADs) are currently the most widely used pharmacological treatment for depression and anxiety disorders. They are employed to reduce the symptoms of low mood, anxiety, and anhedonia, as well as to prevent relapse (National Institute for Health and Care Excellence, 2022; American Psychological Association APA, 2019). Antidepressants are also prescribed for many conditions other than depression, including insomnia, neuropathic pain, smoking cessation and migraine (Lunghi et al., 2022; Wong et al., 2016).

ADs are grouped according to the Anatomical Therapeutic Chemical (ATC) classification system of the WHO with the code N06A (World Health Organization Collaborating Centre for Drug Statistics Methodology WHO, 2021). The group is subdivided according to different modes of action: 1) tricyclic ADs (TCAs and N06AA), 2) selective serotonin reuptake inhibitors (SSRIs and N06AB), 3) monoamine oxidase inhibitors (MAOIs and N06AG), and 4) other ADs (N06AX), which have unique structures and properties that target diverse receptors in the central nervous system, e.g., serotonin norepinephrine reuptake inhibitors (SNRIs) or tetracyclic ADs.

The older ADs include TCAs and MAOIs used to be the main treatment for depression, but they are no longer preferred due to their serious side effects (Montgomery et al., 2007; Thaler et al., 2011; Mandrioli et al., 2012). The new ADs, including SSRIs, SNRIs, and dopamine reuptake inhibitors, have fewer side effects than the older ADs (Sanchez et al., 2014). Although the different classes of new antidepressants have a similar effectiveness on quality of life, they differ in their pharmacokinetics, pharmacodynamics, and side effects, which may impact treatment selection (Cipriani et al., 2009). In addition, the recent increase in the use of SSRIs is related to the increase in their long-term use and the fact that they are increasingly prescribed for conditions other than depression (Montgomery et al., 2007).

The use of ADs has steadily increased since they entered the market. In the US, the use of ADs increased almost 65% between 1999 and 2014 (Pratt et al., 2017). In Europe, AD consumption has doubled between 2000 and 2020 (Lewer et al., 2015). Iceland, Portugal and Sweden are among the top five Organization for Economic Cooperation and Development (OECD) countries with the highest prescription rates for ADs, above 130 defined daily dosage per 1000 inhabitants per day (DID) in 2021, whereas Spain presents a value of 92.7 DID. Latvia has the lowest prescription rate of 21.5 DID (Organization for Economic Cooperation and Development health statistics OECD, 2021).

This increase can be explained by several factors, for example, an increased mental health awareness or individual attitudes towards people with mental health problems (Lewer et al., 2015) and higher number of long-term users. However, this rapid increase in the use of ADs worldwide has prompted discussion about why this increase is occurring and whether ADs are being prescribed appropriately.

While the presence of a diagnosis by a general practitioner is a major reason for the use of ADs, the simultaneous analysis of several and interrelated socio-economic and demographic factors could provide a better picture of the distribution of AD use in a given population. Several studies have reported that factors such as urbanity, percentage of immigrants, gender, population over 65 years, education level, socioeconomic status (e.g., low income and unemployment), geographical area (capital and other areas outside the capital), prices, etc. may explain possible differences in the use of ADs (Morrison et al., 2009; González-López et al., 2015; Mars et al., 2017; Halonen et al., 2018; Eid et al., 2019; Lalji et al., 2021; Oscoz-Irurozqui et al., 2025).

However, a few studies in Europe have focused on local zones such as region, island, municipalities or health areas to establish a dispensation pattern of ADs use. For example, Morrison et al. (2009) examined patterns of regional variation of ADs prescription in primary care in Scotland. Higher levels of prescribing were found in cities and urban areas and lower levels were found in rural areas. Generaal et al. (2019) analyzed the regional differences in ADs prescription in three regions in the Netherlands. The findings suggest that it is not population density in the geography area, but rather the quality of socioeconomic, physical and social geography area characteristics that is associated with the presence and severity of affective disorders.

Lalji et al. (2021) conducted a study of antidepressant prescribing trends in England 2015–2019. Antidepressant prescribing was higher in urban locations (London area) than in rural areas (North West and North East of England). However, socio-economic factors have a complex interplay affecting AD prescribing.

Moreno et al. (2023a) evaluated the distribution of ADs on the island of Fuerteventura in the Canary Islands, a semi-urban area, at the municipal level. Differences in patterns of ADs use are influenced by geographical location (i.e., living in a rural/urban area) and, to a lesser extent, by the type of prescription.

However, the geographical variations have not been studied in such detail over a long period of time, especially in geographically isolated area such as the Canary Islands. In this situation, it is important to determine whether the patterns of consumption are in line with previous studies that show an increase in the dispensing of ADs use and in terms of the specific ADs that are dispensed in accordance with the recommendations of guidelines for the treatment of depression.

This study had two main objectives: firstly, to analyze the use of ADs in the Canary Islands, Spain, between 2016 and 2021, and secondly, to ascertain whether differences in AD use could be attributed to the demographic and socioeconomic characteristics of each island and municipality, as representative of local and isolated geographical areas with urban and rural populations. Furthermore, an investigation was conducted into the utilization of ADs, particularly focusing on the accessibility and prevalence of newer ADs across diverse geographical regions and socioeconomic backgrounds. In this context, a model, based on covariance analysis (ANCOVA) with two nested fixed factors and one co-variable was used to perform contrast analyses.

In order to achieve these objectives, a drug utilization study was conducted in accordance with the ATC Classification and Defined Daily Dose (DDD) methodology (World Health Organization Collaborating Centre for Drug Statistics Methodology WHO, 2021). The data obtained from wholesalers supplying community pharmacies at the population level were utilized as a database.

## 2 Materials

### 2.1 Data

An observational retrospective study was conducted on the use of drugs within the N06A therapeutic group (as classified by the ATC) in an out-of-hospital setting in the Canary Islands, Spain.

The following three subgroups of ADs drugs were considered: (1) N06AA, non-selective monoamine reuptake inhibitors (or tricyclic ADs, TCAs); (2) N06AB, selective serotonin reuptake inhibitors (SSRIs); (3) N06AX, other antidepressants, (other ADs). The dispensation of N06AG, selective monoamine oxidase A inhibitor (MAOIs) was negligible (<0.01%) and only were dispensed on the island of La Gomera.

The present study employed the raw data obtained from wholesalers supplying community pharmacies at the population level in the Canary Islands over the period 2016–2021. The data were supplied by the pharmaceutical cooperatives operating in the Canary Islands. The data set encompasses the entirety of the drug’s distribution within the specified region, as there are no alternative supply channels that could potentially influence the outcomes observed. Only data on ADs dispensed in the pharmacies under prescription were collected.

Additionally, the defined daily dose (DDD) proposed by the WHO for each drug in the study was used to determine the DDD´s per 1000 inhabitants/day (DID) (World Health Organization Collaborating Centre for Drug Statistics Methodology WHO, 2021).

Annual population was defined as the number of residents in a geographic zone at each year on 1st January. These data were extracted from National Statistical Institute (Instituto Nacional de Estadística de España (INE), 2024) and Istac. Data on ADs consumption in Spain were obtained from the National Health System and Pharmacy Authority-Ministry of Health of Spain (Ministry of Health of Spain, 2024).

The final aggregated database used in this study was designed by our research team and has been previously applied in related studies (Moreno et al., 2023b; Oliva et al., 2023). In this context, the database offers data regarding the dispensation of ADs, their annual DID, and the geographical area (province, island, municipality) in which the drug was dispensed.

### 2.2 Sociodemographic characteristic and population in the Canary Islands

**FIGURE 1:**
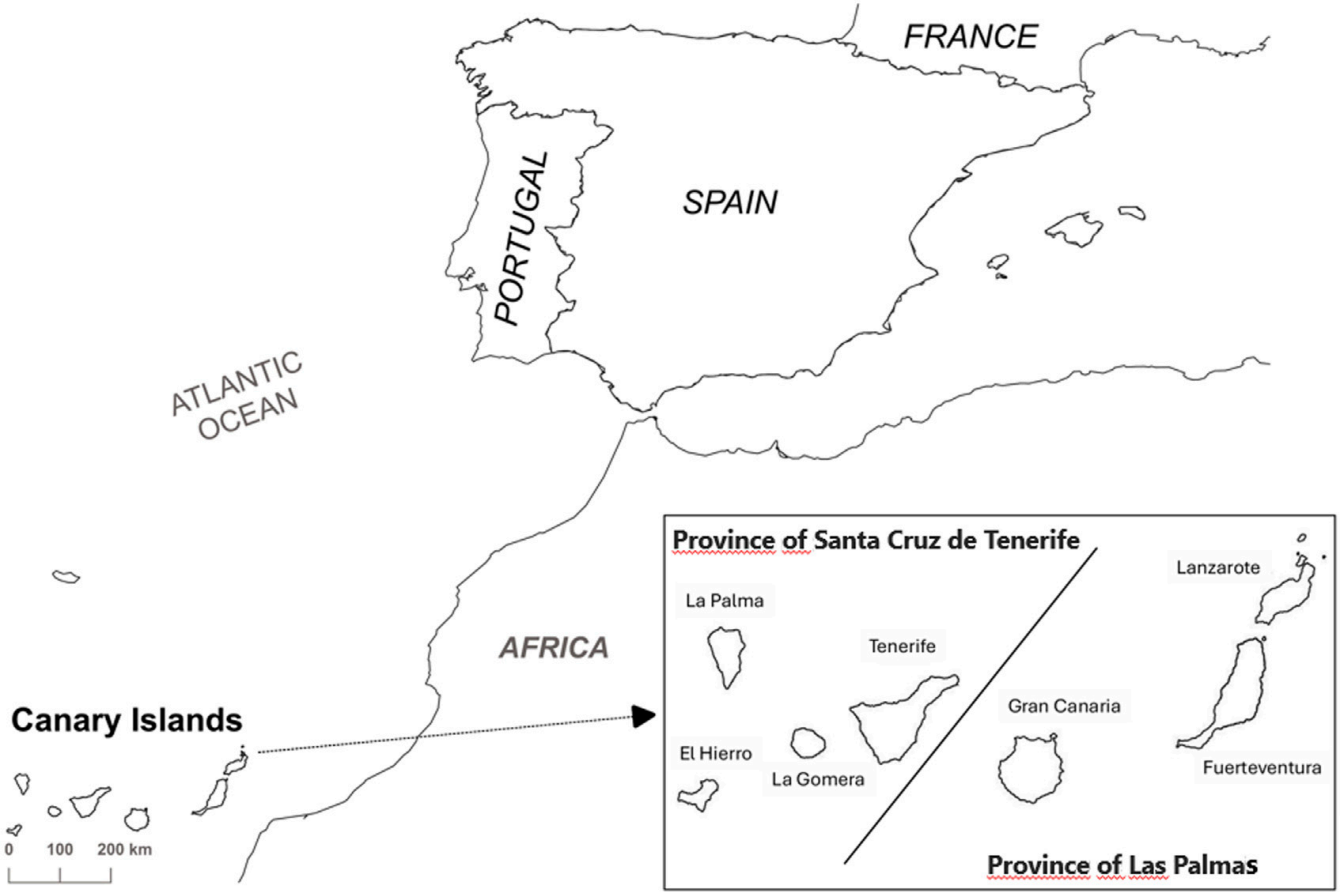
Map of the Canary Islands with the two provinces and islands that make up each one.

Figure 1 shows the map of the Canary Islands, Spain, with the two provinces, Las Palmas and Santa Cruz de Tenerife and the islands that make up each province. Table 1 shows the population and demographic data at the provincial and island level. During the analyzed period, the population at the province of Las Palmas increased by 2.7%, whereas the increase by islands was different, an 0.9% for Gran Canaria and 7.1% and 10.1% for Lanzarote and Fuerteventura, respectively. In addition, an increase of 3.86% was observed in the province of Santa Cruz de Tenerife, whereas this increase varied between 2.3% for La Palma and 6.3% for El Hierro.

**TABLE 1:** Demographic and socioeconomic data for 2021 years and its variation (∆), expressed as a percentage obtained during the period 2016–2021, for different islands and both provinces in the Canary Islands. Data obtained from National Statistics Institute in Spain (INE) and Istac.

Island	Population	∆(%)	Population density	∆(%)	Over-65 years (%)	∆(%)	Income (€/year)	∆(%)
Gran Canaria	852,688	0.9	547	0.9	16.5	11.2	11,550	13.4
Lanzarote	156,189	7.1	185	7.0	11.9	−7.6	10,375	9.3
Fuerteventura	119,662	10,1	72.0	10,8	9.7	19.8	9,818	7.6
Province of Las Palmas	1,112,539	2.7	277	2.7	15.8	10.8	11,172	12.3
Tenerife	927,993	3.97	456	3.97	17.0	8.57	11199	17.3
La Palma	83,380	2.27	118	1.95	21.2	3.45	11046	19.9
La Gomera	21734	3.65	58.9	3.65	22.3	3.50	11,543	17.8
El Hierro	11,298	6.29	42.2	6.31	23.0	3.52	11,630	19.2
Province of S/C de Tenerife	1,044,405	3.86	309	3.85	17.5	7.87	11198	17.5

However, Gran Canaria and Tenerife are considered as urban areas (Reig et al., 2016), with more than 40% of the population in the capitals, which are home to the regional administration buildings, the reference hospitals and other essential services. La Palma, Lanzarote and Fuerteventura are considered as semi urban areas, but differ slightly. Fuerteventura has the lowest population density and the smallest proportion of elderly people (9.72%), while 41.5% of Lanzarote’s population lives in the capital. La Palma is similar in this respect, but the proportion of elderly people is twice that of both islands. El Hierro and La Gomera are the smallest and least populated islands but with most elderly proportion of the population (23%). Both are considered as rural areas.

At the municipal level, El Hierro has only three municipalities, while La Gomera, Lanzarote and Fuerteventura have six and La Palma fourteen. Gran Canaria and Tenerife, the main islands, have twenty-one and thirty-one municipalities, respectively. However, all the islands have urban and/or semi-urban and rural municipalities, with the exception of La Gomera and El Hierro, which are all considered rural (Reig et al., 2016).

### 2.3 Statistical analysis

The following statistical model was used for analysis at island level:

$$y_{ij}=\left(\beta_{0}+\alpha_{0,i}\right)+\left(\beta_{1}+\alpha_{1,i}\right)*X_{ij}+\varepsilon_{ij}$$ (1)

where y_ij_ is the response for the island xj -th for the *i*th time; β_0_ and β_1_ correspond to the intercept and slope of the reference island; α_0,i_ are the differences between intercepts for island 1 and island 2, 3, . . . k, and α1,i are the differences in the slopes. The term ε represents the error associated with the response *j*th measured on the *i*th time.

At municipality level, the following model based on covariance analysis (ANCOVA) with two nested fixed factors and one co-variable was used to analyze variation of DID during the study period:

$$y_{ij}=\mu +A_{i}+B_{j,i}+\beta_{0}A_{i}+\beta_{1}x_{i}+\epsilon_{ij}$$ (2)

where y_ij_ is the dependent variable (i.e., response) and x_i_ is the time (in years). The two factors used were: A_i_ is the effect of the *i*th island (i = 1 … 7) and B_j,i_ is the effect of the *i*th municipality within the *i*th island (j = 1.5 for i = 1; j = 1 .7 for i = 2; j = 1 … 21 for i = 3; j = 1.3 for i = 4; j = 1.6 for i = 5; j = 1.13 for i = 6; j = 1.31 for i = 7) and Є_ijk_ is the error term assuming it is independent following a normal distribution with zero mean and constant variance (N ~ (0, σ2)). In order to analyze the variation of the drug’s prescription over time, the interaction “island x time” was included in the model. All of these calculations were performed using different functions and libraries available in the free R-program, version 4.6.2 (R Core Team, 2021) (www.r-project.org).

In addition, it is easy to extend this model to include m factors. In this context, the median household income was used to measure the socio-economic level of each municipality and island, while the percentage of the population aged over 65 years and the population density were used as socio-demographic variables. All these data were downloaded from a public demographic database (Instituto de Estadística de Canarias, 2024).

### 2.4 Ethics approval

Patient/subject data such as age, sex or pathology were not provided, and therefore the report of the Ethics Committee of the University of La Laguna or the informed consent of the patient/subject were not required. Data analysis was performed according to the relevant guidelines and regulations.

## 3 Results

### 3.1 Data analysis at the province and island level

Table 2 shows the percentage in the variation of the overall DID during the study period at the provincial level and for each island. Overall, we observed a significant increase in ADs consumption throughout the Canary Islands between 2016 and 2021, a value of 30% was observed. A similar trend was observed at both provinces, although the increase was higher in the province of Las Palmas 32.81% against 27.25% in the province of Santa Cruz de Tenerife. At the level of the islands, the increases ranged from 12.93% in La Palma to 35.22% in Gran Canaria (see Table 2), whereas the increase in population was lowest.

**TABLE 2:** Overall annual DID for each island, Spain as a whole and the variation in percentages during the period 2016–2021.

Island	2016	2017	2018	2019	2020	2021	∆ (%)
Tenerife	64.86	67.62	72.4	75.7	79.62	83.7	29.05
El Hierro	31.55	32.56	34.57	37.34	42.63	42.62	35.09
La Gomera	52.29	54.51	51.05	54.96	59.7	63.48	21.40
La Palma	89.44	91.11	97.14	99.14	100.5	101.9	13.93
Gran Canaria	76.84	81.54	86.35	90	97.08	103.9	35.22
Lanzarote	32.36	34.17	35.43	36	38.84	41.21	27.35
Fuerteventura	30.15	30.11	31.76	33.84	34.69	37.43	24.15
Spain^a^	75.47	77.09	79.58	82.52	86.19	91.51	21.25

^a^ Data for Spain were provided by Spanish Agency of Medicines and Medical Devices (2016–2021).

**FIGURE 2:**
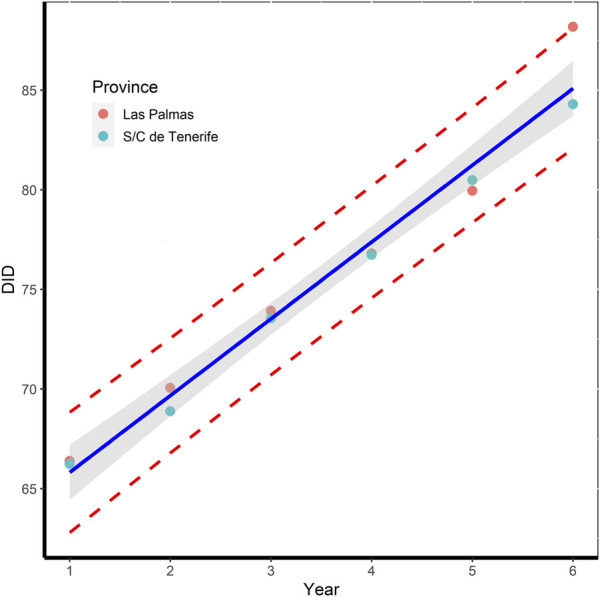
Variation in total DID per year for each province over the period 2016–2021, together with the 95% prediction bands (dashed red line) and the estimated regression line (in blue) with 95% confident limits (grey shaded area).

However, the variation of annual DID was similar for both provinces (see Figure 2), presenting the same estimated dispensation level 61.96 DID and variation rate 3.87 DID per year, although the value was above the expected one in 2021 for the province of Las Palmas, according to the expected evolution of DID in the period 2016–2020. However, the observed value in DID for 2021 (88.17 DID) is within the 95% prediction intervals for the estimated value. The presence of potential outliers was discarded by the Grubb’s test result. The discrepancy observed falls within the range of variability derived from the proposed model, with no anomalous causes to explain the finding. A comparable situation was observed by Moreno et al. (2023b) in the period preceding the onset of the COVID-19 pandemic, which did not alter the overall trend per year in this period.

At the same time, overall DID values at the national level (i.e., Spain as a whole) are on average around 7 points higher than overall DID values at the provincial level, although this difference has halved in the last year. The increase in this period was 21.25% whereas the population only increased by 2.06%. The estimated dispensation level was 71.0 DID [67.72–74.29], whereas the national consumption rate was 3.23 [2.39–4.08] DID per year, approximately, 20% lower than that at the province level (AEMPS, 2024).

All islands presented the same pattern than those observed at province level, exhibiting an increase significant linear in AD consumption. Table 3 shows the results of ANOVA, indicating that the dispensation level (i.e., intercept in the model) and variation rate (i.e., slope) depends on each island. La Palma presented the highest dispensation level (87.27 DID), it was even higher that those observed at provincial level, by approximately twenty-five points, whereas El Hierro, Lanzarote and Fuerteventura had lower values (Table 3). However, the consumption rate was 2.59 DID per year at El Hierro, approximately one and half times higher than that of the other two islands (1.49 DID per year). Gran Canaria presented a dispensation level of 70.74 DID, against 61.96 for Tenerife, whereas the variation rate was higher in Gran Canaria compared to Tenerife 5.30 vs. 3.87 DID per year.

**TABLE 3:** Results of ANOVA for the factors island and island x year for the overall DID. The residual standard error was 1.38 on 32 degrees of freedom.

Source of variation	df	Sum of square	Mean square	F	Pr>(|t|)
Year	1	1204.0	1204.0	631.4	<0.001*
Island	7	26574.7	3796.4	1991	<0.001*
Year x Island	7	205.2	29.3	15.37	<0.001*
Residuals	32	61.0	1.9		

Coefficients	Estimate	2.5%	97.5%	Pr>(|t|)
Intercept^a^	61.96	59.35	64.58	<0.001*
Year^a^	3.86	3.19	4.53	<0.001*
El Hierro	−33.92	−37.62	−30.22	<0.001*
Fuerteventura	−34.19	−37.89	−30.49	<0.001*
Gran Canaria	8.77	5.06	12.47	<0.001*
La Gomera	−13.51	−17.21	−9.81	<0.001*
La Palma	25.31	21.61	29.02	<0.001*
Lanzarote	−31.51	−35.21	−27.80	<0.001*
Tenerife	−1.31	−5.02	2.39	**0.475**
Year x El Hierro	−1.34	−2.29	−0.38	<0.01*
Year x Fuerteventura	−2.37	−3.32	−1.42	<0.001*
Year x Gran Canaria	1.44	0.49	2.39	0.0041 *
Year x La Gomera	−1.70	−2.65	−0.75	<0.001*
Year x La Palma	−1.21	−2.16	−0.26	<0.014*
Year x Lanzarote	−2.18	−3.13	−1.23	<0.001*
Year x Tenerife	−0.056	−1.01	0.89	**0.905**

^a^ Data refer to Canary Islands as a whole (reference level). (*) Significance for p < 0.05.

The dispensation rate would be expected to rise as the number of elderly people (expressed as a percentage of the population aged over 65) increases. The results show that there is low-middle correlation between the variation of overall DID and this variable (r = 0.34). A similar result was obtained for the other two variables: population density (r = 0.58) and median household income (r = 0.45). In this context, demographic and socio-economic characteristics were included in the model described by Equation 1 as factors such as income (€/year), percentage of over sixty-five-years old in the population, population density and interaction term. However, all these new terms were not statistically significant (p > 0.05) and the simpler model was used.

### 3.2 Data analysis by antidepressants drug and classes

Table 4 shows the mean consumption (expressed as percentages) of different AD classes according to the ATC classification for each island and in Spain as a whole during the study period. N06AB subgroup (i.e., SSRI) was the most consumed in the Canary Islands as a whole, with a mean contribution of 61.87%, but with a negative tendency, −1.19 [-1.80; −0.57] % per year, followed by subgroup N06AX (i.e., other ADs), with 34.54%, showing a positive tendency, +1.16 [0.57; 1.76] % per year, and finally the N06AA subgroup (i.e., TCAs) with 3.58% in the study period and stable use. The SSRIs were also the most consumed drugs, regardless of the island, although the level of dispensation (i.e., intercept), and consumption rate of both subgroups depends on each island. For example, Lanzarote presented the highest level for SSRIs, 70.53% against 52.41% for La Palma. A similar situation was observed for the “other ADs” subgroup.

**TABLE 4:** Results of ANOVA for the factors of therapeutic subgroup and island (expressed as a percentage of overall DID).

Subgroup	N06AA	N06AB	N06AX
Intercept^a^	3.58	61.87	34.54
Year^a^	0.0246	−1.19	1.16
El Hierro	5.49	57.19	37.34
Fuerteventura	5.47	67.68	26.85
Gran Canaria	3.58	61.87	34.54
La Gomera	5.82	61.87	32.35
La Palma	2.99	52.41	44.60
Lanzarote	4.58	70.53	24.89
Tenerife	3.58	61.87	34.54
Year x El Hierro	2.46 · 10^−2^	−1.19	1.16
Year x Fuerteventura	2.46 · 10^−2^	−2.62	2,61
Year x Gran Canaria	2.46 · 10^−2^	−1.19	1.16
Year x La Gomera	2.46 · 10^−2^	−0.17	0.26
Year x La Palma	2.46 · 10^−2^	−1.19	1.16
Year x Lanzarote	−0.11	−1.19	1.16
Year x Tenerife	2.46 · 10^−2^	−1.19	1.16

^a^ Data refers to Canary Islands as a whole (reference level).

The consumption rate of the SSRI subgroup was higher for the island of La Gomera and Fuerteventura, with negative tendency, but at different rate, a decrease of −2.62% per year being observed for Fuerteventura against −0.17% per year for La Gomera, whereas in the “other ADs” subgroup consumption increased by the same magnitude (+2.61% per year) in Fuerteventura while for La Gomera increased 0.26% per year (see Table 4). For the rest of the islands, there are no differences between them with respect to the consumption rate of SSRIs, with a decrease of 1.19% per year being observed, whereas in the “others ADs” subgroup consumption increased by the similar magnitude (+1.16% per year). In this case, all the differences between terms were statistically equal, since the 95% confidence interval for the mean difference between two terms included the zero (data not shown). The data in Spain as a whole shows the same change in tendency but at a lower rate, −0.86 [-1.03, −0.69] % per year, three times lower than those observed in La Gomera and Fuerteventura, but lower than those of the rest of islands.

In contrast, the consumption of TCAs remained fairly stable in all islands over the last 5 years, although the dispensation level was different, 5.5% on average in La Gomera, Fuerteventura and El Hierro versus 2.99% for La Palma. Lanzarote was the exception presenting a negative variation rate of −0.11 [-0.27; −1.4·10^−3^] % per year.

The next step was to examine the trends in prescription of ADs drugs on all islands in order to determine whether there are differences between them. Table 5 shows the variation in DID for first five more consumed active pharmaceutical ingredients (API), expressed as percentages with respect to the annual DID, for each island. Sertraline ranks first, followed by escitalopram and paroxetine, except Lanzarote where escitalopram was ranked first followed by sertraline. The other two positions correspond to other SSRIs, normally fluoxetine or belonging to the “other ADs” subgroup such as venlafaxine, desvenlafaxine, duloxetine or mirtazapine. It should be noted that, of the first four APIs, at least three belong to the SSRI subgroup while the fourth corresponds to the “other ADs” subgroup.

**TABLE 5:** Variation of DID by API (expressed as a percentage of overall DID) during the period 2016–2021 for each island.

Tenerife	El Hierro	La Gomera	La Palma
Sertraline (21.31%)	Sertraline (15.87%)	Sertraline (19.33%)	Sertraline (21.40%)
Escitalopram (16.05%)	Escitalopram (15.64%)	Paroxetine (17.31%)	Desvenlafaxine (17.02%)
Paroxetine (9.82%)	Paroxetine (9.90%)	Escitalopram (15.15%)	Escitalopram (10.12%)
Duloxetine (8.20%)	Duloxetine (9.68%)	Venlafaxine (8.76%)	Duloxetine (9.12%)
Desvenlafaxine (7.34%)	Fluoxetine (8.01%)	Duloxetine (8.56%)	Fluoxetine (8.89%)
Others (37.28%)	Others (40.90%)	Others (30.89%)	Others (33.45%)

Gran Canaria	Fuerteventura	Lanzarote
Sertraline (17.05%)	Sertraline (17.25%)	Escitalopram (21.40%)
Escitalopram (14.80%)	Escitalopram (12.08%)	Sertraline (17.02%)
Paroxetine (11.12%)	Paroxetine (11.44%)	Paroxetine (9.69%)
Venlafaxine (7.54%)	Fluoxetine (9.82%)	Fluoxetine (9.28%)
Fluoxetine (7.40%)	Mirtazapine (6.99%)	Citalopram (6.30%)
Others (42.09%)	Others (42.42%)	Others (36.31%)

### 3.3 Data analysis at the municipality level

The variability in overall DID between-islands and municipalities during the study period was analyzed using an ANCOVA according to the model proposed in Material and Methods, using the Equation 2 (Table 6). The coefficient of adjusted determination (R^2^) was 0.955, indicating that the model proposed is suitable for data interpretation. Furthermore, the inclusion of demographic and socio-economic variables in the model resulted in an increase in the coefficient of determination (R^2^) up to 0.963. However, these additional terms were not statistically significant (p > 0.05), and thus the simpler model was deemed to be more appropriate. The null hypothesis for both the island factor and the interaction term “island x year” were rejected (p < 0.05), indicating that the overall DDD varied between islands during the study period. This finding was corroborated by Tukey’s test (p < 0.01). In addition, the overall DID variation rate varied between-islands as indicated in the term “island x year” (Table 6). In this case, Tenerife, El Hierro, La Gomera and Fuerteventura had the same estimated dispensation level (32.38 DID), whereas the estimated variation rate was different with Tenerife at the front (3.98 DID per year) and approximately half of that at El Hierro and Fuerteventura (2.04 DID per year). Gran Canaria and La Palma showed the highest dispensation levels (73.22 and 72.27 DID, respectively), but the same variation rate as Tenerife. However, Lanzarote presented the lowest dispensation level (17.67 DID) with a variation rate of 2.60 DID per year, 1.5 times lower than Tenerife.

**TABLE 6:** Results of ANCOVA according to the two nested factor model for the overall DID. The residual standard error was 6.35 on 423 degrees of freedom.

Source of variation	df	Sum of squares	Mean square	F	Pr<|t|
year	1	17212	17212	427.6	<2·10^−6^*
island	6	161548	26924.6	618.8	<2·10^−6^*
year x island	6	1886	314.3	7.81	<2·10^−6^*
island x municipalities	79	263091	3330.3	82.7	<2·10^−6^*
residuals	423	17028	40.3		

Parameter	Estimate	2.50%	97.50%	Pr (>|t|
Tenerife^a^	32.38	26.95	37.80	<2·10^−6^*
El Hierro	−4.88	−14.45	4.69	**0.317**
Fuerteventura	−0.007	−8.79	8.78	**0.999**
Gran Canaria	40.84	33.06	48.62	<2·10^−6^*
La Gomera	−5.13	−13.71	3.44	**0.240**
La Palma	39.90	31.91	47.88	<2·10^−6^*
Lanzarote	−14.71	−23,13	−6.28	0.001*
Year^1^	3.94	3.40	4.47	<2·10^−6^*
El Hierro x year	−2.03	−3.83	−0.22	0.028*
Fuerteventura x year	−2.04	−3.48	−0.60	0.005*
Gran Canaria x year	0.54	−0.30	1.38	**0.207**
La Gomera x year	−2.63	−3.96	−1.3	<2·10^−6^*
La Palma x year	−0.68	−1.66	0.31	**0.175**
Lanzarote x year	−2.61	−3.85	−1.36	<2·10^−6^*

^a^ Data refer to Tenerife (reference level). (*) Significance for p < 0.05.

Furthermore, the data analysis from Table 6 indicates that there are differences between municipalities from different islands (i.e., island x municipality term) but also within the same island (data not shown). In order to confirm this result, a second analysis was performed to determine the possible differences between municipalities for each island as well as their evolution during this period, and the marginal means were calculated to do this (Supplementary Table S1). For example, the municipality of El Pinar, on the island of El Hierro, differs significantly with respect to the other two municipalities on the island. A similar situation was observed for the municipalities of Agulo, in La Gomera, and Tias in Lanzarote, where all pairwise differences are statistically significant (p < 0.05). The situation in Gran Canaria and Tenerife is more complex since each has thirty-one and twenty-one municipalities, respectively and therefore, the contrast analysis shows more than 200 pairwise entries. However, the results show differences between municipalities with different levels of urbanity, but there are also cases where a rural area has a high consumption similar to that of an urban area, such as the case of Santa Cruz de Tenerife, capital of the island of Tenerife, a mainly urban area, and La Guancha, a rural area, with similar marginal means of 109.8 and 103.1 DID, respectively. Arona, Adeje and San Miguel de Abona, considered as touristic municipalities, located in the south of the island, presented different marginal means from 19.5 DID for San Miguel de Abona to 56.9 DID for Arona. The smaller municipalities with less than 2,000 inhabitants and a disperse population, low socioeconomic status and generally with a greater proportion of elderly people (>20%) show the same trends with marginal means varying between 45.5 DID for Santiago del Teide and 70.9 DID for Vilaflor de Chasna.

A similar situation was observed for Gran Canaria. In this context, Las Palmas de Gran Canaria, capital of the island, and Telde, urban areas, present a consumption of 94.8 and 97.3 DID, respectively, whereas Mogán and San Bartolme de Tirajana, two touristic municipalities and also urban areas, show half the consumption of the capital. However, municipalities such as Moya, Galdar, Santa María de Guía or Ingenio, with approximately 20,000 inhabitants each, had DID values above 100, more than 25 points with respect to the annual DID at the island level. In contrast, Artenara and Tejeda, with less than 2,000 inhabitants each, and the highest proportion of elderly people (>30%), had a lower DID (22.9 and 30.3 DID). The main discrepancy was observed in La Palma between two neighboring municipalities, Breña Alta and Breña Baja, with a difference of 116.7 DID.

## 4 Discussion

### 4.1 Consumption at provincial and island level

The aim of our study was to provide data on the patterns of ADs use in the Canary Islands from 2016 to 2021, using an aggregated database through dispensed prescriptions. Although other studies have analyzed the consumption of ADs over time and evaluated its trends, our analysis is the first at the level of a geographically isolated area.

Overall, we observed a significant increase in ADs consumption throughout the Canary Islands between 2016 and 2021, a value of 30% was observed, whereas in Spain as a whole was 21.25% in the same period. At the level of the islands, we can find values that are either below or above the national average, such as those of La Palma and Gran Canaria (see Table 2).

The results of our study are similar to those observed in other European and OECD countries, an average increase rate of 32.5% was observed, whereas in the EU was lower, 19.83%, with increases ranging from 3% in the Netherlands and Switzerland to 59% in Finland (Peano et al., 2010).

In addition, all the islands showed the same pattern as that observed at provincial level, with a significant linear increase in AD consumption. However, the level of dispensation and the rate of variation vary from one island to another (see Table 3).

Most European countries showed a significant linear increase (Hungary, Luxembourg and Sweden), although plateauing (the Netherlands and the United Kingdom) and exponential growth (Portugal and Sweden) were also observed.) Denmark showed a downward trend over time, while France and Norway had stable consumption levels. The level of dispensation and the rate of variation were also different. In this case, Hungary and Luxembourg had the lowest rate of variation, 0.36% per year, while Sweden and Iceland had the highest, 2.97% and 5.49% per year, respectively (Peano et al., 2010).

Our results are in line with those observed in European countries. However, there are some differences. The results show islands with a consistently higher consumption than the average of the countries (La Palma, Gran Canaria and Tenerife), while the rest of the islands, the consumption was half than the average, except for La Gomera with an average consumption similar. La Palma, Lanzarote and Fuerteventura have variation rates slightly below the average (1.49% vs. 1.68% per year), while Gran Canaria has a rate similar to that of Iceland.

These differences in prescription patterns could be related with the geographic area itself, its demographic characteristics and its economy activity which is closely linked to tourism and/or the primary sector. Lanzarote and Fuerteventura, with similar sociodemographic characteristics and economies, based on tourism, presents the same trends. However, the two smallest islands have different consumption rates since EL Hierro has a variation rate higher than La Gomera but with lower DID levels, 28.04 vs. 48.45 DID. Both islands are considered as rural areas with agriculture as the main activity economic and the highest proportions of elderly people in the Canary Islands, although the geography location of the population is different. Approximately, 60% of the population in La Gomera is concentrated in the capital and Valle Gran Rey municipality with a high socioeconomic status with easy access to all essential services, whereas the population in the smaller municipalities is highly disperse, with low socioeconomic status, a lower population density (on average, 40 inhabitants per km2), characterized by small villages, far away from essential services and with a high proportion of elderly people (Agulo and Vallehermoso have values above 30% against 21.4% on average).

EL Hierro has half the population with respect to La Gomera with a density of 40 inhabitants/km2, where 45.5% of the population live in the capital, Valverde, while El Pinar is the smallest municipality and least populated (17.5%), with a population that is not spread out and with easy access to the capital. The three municipalities have a low socio-economic status, with agriculture being the main economic activity and the oldest population in the Canary Islands, with an average of 22.3%.

The two main islands of Gran Canaria and Tenerife considered as urban areas have comparable patterns, although consumption was slightly higher in Gran Canaria than in Tenerife. The average difference in the period analyzed was 5.5%, despite the smaller population of Gran Canaria, where the population increased by 0.9% compared to 4% in Tenerife. In this context, Geographical location and the size of the population living in the capital are the main reasons for the difference. In Gran Canaria, 44.41% of the population lives in the capital, compared with 24.46% in Tenerife. The tourism and services sector are the main economic activities (80%) in both islands, but the unemployment rate is different, 22% in Gran Canaria, six points higher than in Tenerife (data on 2022 years). As a result, there is a larger population accessing and needing health services.

La Palma is a semi-urban area with a strong focus on the primary sector (banana production and cultivation). Tourism also plays a role, although to a lesser extent than in Lanzarote. However, La Palma has the highest consumption of all the Canary Islands (87.28 DID), three times higher than Lanzarote, with half the population but twice the proportion of elderly people (21.2% vs. 11.9%).

Therefore, the results show differences between islands with different levels of urbanity. The increase was higher in La Palma, considered as a semi-urban area, followed by Gran Canaria and Tenerife, urban areas. La Gomera, with 23,000 inhabitants, is a rural area. It has a similar dispensation level to Tenerife, while El Hierro is 20 points below the value of Tenerife. Lanzarote and Fuerteventura, considered as semi-urban areas, have the lowest dispensation levels.

This result differs from that described by Morrison et al. (2009), where AD dispensing in Scotland was higher in urban than rural areas. Lalji et al. (2021) analyzed the AD prescribing trends in England between 2015 and 2019. Theses authors obtained the same conclusions, although the AD prescribing was higher in more deprived areas.

Halonen et al. (2018) examined the geographical and educational differences in the use of ADs in Finland. These authors show that the use of ADs was higher in the capital compared to all other areas and among those with high versus low education. These differences in AD use suggest socioeconomic inequalities in the quality of mental health treatment.

In contrast to the above studies, Generaal et al. (2019) used a cross-sectional study to show that socioeconomic, physical and social characteristics, rather than urbanization degree, are associated with the presence and severity of depression and anxiety.

Gómez-Lumbreras et al. (2019) describe the use of ADs across in five European settings, with different economic, sociodemographic and cultural characteristics. AD use has risen in all the studied settings. Apart from demographic and economic conditions, cultural patterns may play a role on prescribers and may influence the overall AD use.

### 4.2 Consumption by antidepressant drug and class

The geographical variations in the utilization of different ADs classes have not been previously examined in such detail over an extended study period, particularly in geographically isolated areas. In this point, SSRIs were the ADs most commonly used, followed by the “other ADs”, whereas the consumption of TCAs remained stable during the study period. Furthermore, the variations observed for the different APIs did not differ significantly during this period on all the islands. The ranking of different AD classes was fairly stable on each island and year, although the percentages and ranking varied slightly. In addition, this result is consistent with other national data and comparable with those reported in other European countries (Martín-Arias et al., 2010; Hernandez et al., 2012; Gualano et al., 2014; González-López et al., 2015).

However, Lipovec et al. (2022) show that SSRI use in Slovenia decreased from 68.0% to 58% between 2009 and 2018. Similar results were found by Luo et al. (2020) in the United States, where SSRI use decreased by 3.8 points between 1996 and 2015 (68.0%–64.2%).

The consumption for the “other ADs” subgroup has increased in the last years, from 23.5% in Sweden to 43% in Denmark, whereas in Norway it was stable, whereas the TCA use was fairly stable in the majority of European countries, less 10% of the overall use, except Italy, where a decrease of 21.4% during the period 2000–2011 was observed, although this trend has recently been confirmed by Oscoz-Irurozqui et al. (2025), with a decrease of 15.62% over the period 2008–2019, with values remaining consistently below 2% and a variation of −1.46% per year.

The Nordic countries also showed a decrease in the consumption of TCA, ranging from 2.9% in Norway to 5.1% in Sweden. In contrast, Germany and the Netherlands show a change in tendency, approximately 26.5% of TCAs were prescribed in Germany (Bauer et al., 2008; Parabiaghi et al., 2011; Gómez-Lumbreras et al., 2019).

Brauer et al. (2021) analyzed the consumption of ADs with the three categories in 65 countries and regions during the period 2008–2019 using a longitudinal study. Globally, a relative average increase per year of 3.50% was observed but with a great variability among countries in the dispensation level. In addition, an increase in the consumption of SSRS and “other ADs” was observed, whereas the consumption of TCAs decreased during the study period.

Similar results have been obtained in different Spanish regions. Serna et al. (2010) conducted an observational transversal study on the use of ADs in Lleida, one of the provinces of Cataluña, between 2002 and 2007. They report that SSRIs were the most consumed, followed by the subgroup “other ADs”. This result was confirmed by Gómez-Lumbreras et al. (2019) for the period 2007 to 2011. The use of ADs in Valencia, Spain, was analyzed by Sempere Verdú et al. (2014) during the period 2000–2010. The SSRIs were the most prescribed with 63.9% of prescriptions. In Castilla y Leon, the use of SSRIs had increased by up to 77% at the end of 2005 (Martín-Arias et al., 2010). Gutiérrez-Abejón et al. (2020) have also analyzed the use of ADs in the above Spanish region, during the period from 2015 to 2018. These authors point out that the SSRIs and “other ADs” were the most commonly used. However, this study was not carried out on the resident population of this geographical area, which limits the conclusions drawn. In all the studies, the main conclusion was an increase in the dispensation of SSRIs and “other ADs” groups, while there was a reduction in the dispensation of TCAs. In this regard, SSRIs have largely supplanted TCAs due to their superior tolerability and safety profiles.

The data expressed in DID provide precise information on the use of prescription ADs. However, the problem could be analyzed from another perspective, for instance, by examining the distribution of each subgroup as a percentage of the overall DID. This approach allows for the monitoring of the evolution of these subgroups over time in a specific geographical area. This approach enables the data to be normalized and comparisons to be made between islands.

A decrease in SSRI consumption was observed from the year 2016, but at the same time an increase of the same magnitude was found in the “other ADs” subgroup, which was higher in Fuerteventura (2.61% DID per year) compared to La Gomera (0.26% DID per year), whereas the consumption of TCAs was low and stable (see Table 4), with values of 2.99% in La Palma and slightly higher ones in Gran Canaria and Tenerife (3.58%). The rest of the islands have values above 5%. The data obtained indicate that all islands exhibit a similar pattern of AD prescription, although with varying levels of prescription and consumption rates. At the national level, the prescriptions for both subgroups were comparable to those observed in the Canary Islands as a whole. Additionally, both showed a similar change in tendency to those observed on the islands, but at a lower rate (0.86% DID per year). The authors believe that population characteristics and size could explain this difference.

However, in our study, the TCA consumption remained stable and above 5%, especially in El Hierro and La Gomera. In this context, TCA are prescribed for indications other than depression or anxiety, particularly in chronic pain and sleep disorders. In this point, both islands have the most elderly proportion of the population with 23% on average, although various municipalities have levels higher than 30% in La Gomera and population very disperse. The introduction of these new therapeutic options could justify their increased use in this antidepressant category and thus be one of the possible reasons for the change in trend seen in our analysis compared to other geographical areas.

Despite the large number of studies carried out in Europe on the use of ADs, most of them only provide data on the variation in overall DID over time, without providing information on the contribution made by each therapeutic subgroup to the overall DID. In this regard, the works of Gualano et al. (2014), González-López et al. (2015) and Gómez-Lumbreras et al. (2019) should be highlighted because they provide information on this contribution in several European countries and two Spanish regions.

The published results show that the level of contribution and the trends in the different therapeutic subgroups have not changed in recent years, with SSRIs being the main contributors to overall DID with a negative tendency, followed by the subgroup of “other ADs” and with a positive tendency, while the use of TCAs has been stable or decreasing. The exception was Norway. In the case of the two Spanish regions, data and patterns were similar to other European countries. However, the differences observed between them could be linked to sociodemographic, cultural and economic factors (Loyola Filho et al., 2014; Lewer et al., 2015; Gómez-Lumbreras et al., 2019).

The AD prescribing patterns and factors influencing the choice of ADs for the treatment of depression were examined by Bauer et al. (2008) in 12 European countries using a prospective observational study. Physical factors (age, gender) and patient factors (severity of depression, age, education, smoking, number of current physical conditions and functional syndromes) were associated with initial AD choice, whereas for the new episode of recurrent depression, the choice of the AD prescribed was strongly influenced by the previous use of ADs and classes.

The analysis of SSRIs reveals an increase in the consumption of sertraline, escitalopram, paroxetine and fluoxetine (Table 5) on all the islands. All these APIs are indicated for the treatment of major depressive disorder, anxiety and social anxiety disorders. These findings are consistent with those reported in other regions of Spain and in European countries, although the ranking of the substances differs slightly. A series of studies conducted in the United Kingdom and the Netherlands have demonstrated that escitalopram constitutes the most consumed substance, whereas paroxetine consumption has either stabilized or declined. The consumption of sertraline in the United Kingdom has doubled over the course of the pandemic, as evidenced by two studies (Lalji et al., 2021; Rabeea et al., 2021). In the subgroup of other ADs, the most recent medications, including desvenlafaxine, mirtazapine, venlafaxine and duloxetine, were the most consumed.

The ranking of different APIs was fairly stable on each island and year, although the percentages and ranking varied slightly. For example, on the island of Lanzarote, escitalopram was the most consumed followed by sertraline, whereas sertraline was the most frequent in the rest of the islands with percentages varying between 21.4% in La Palma and Tenerife versus 15.87% in El Hierro. In the province of Santa Cruz de Tenerife, duloxetine was the most commonly used drug, whereas in the other province, venlafaxine and mirtazapine were the most commonly used drugs in the subgroup ‘other ADs’. This difference may be explained by general medical practice. Gran Canaria has two reference hospitals in the capital. Patients from Lanzarote and Fuerteventura are referred to them according to their place of residence and not according to their illness. The same is found in the other province, with Tenerife as the center of reference.

The American Psychological Association and the National Institute for Health and Clinical Excellence (NICE) recommend pharmacotherapy as a treatment for major depression (National Institute for Health and Care Excellence, 2022; American Psychological Association APA, 2019). Pharmacotherapy typically involves the administration of an AD, which is most commonly an SSRI, such as sertraline, escitalopram, or paroxetine. In the event that the response to the optimal doses of one of the SSRIs is inadequate or poorly tolerated, it is recommended that a different SSRI be considered. In the event that there is no improvement after 8–12 weeks, it is recommended that another drug with a different mechanism of action be considered, such as venlafaxine, desvenlafaxine, duloxetine or mirtazapine. The results from the Canary Islands are therefore fully in line with the recommendations of depression treatment guidelines.

### 4.3 Consumption at municipal level

The variability between islands and municipalities was analyzed by using an ANCOVA. The results of the approach confirm the observed differences between the islands, as well as the variation in DID over time between the municipalities of the islands (see Table 6). In addition, the estimated dispensation level was the same in four of the seven islands (Tenerife, El Hierro, La Gomera and Fuerteventura), while Gran Canaria and La Palma had the highest levels, nearly four times higher than in Lanzarote (73.22 versus 17.67 DHD) (see Table 6). However, Gran Canaria, Tenerife and La Palma have the highest variation rate (3.94 DID per year), which is double that of Fuerteventura and El Hierro, which are considered equal. The lowest rates (see Table 6) are found in Lanzarote and La Gomera.

However, these results are not consistent with those obtained using annual DID data. The main discrepancy is found in the dispensation level, where the values are markedly different. Lanzarote stands out with a level of 17.67 DID, which is about half of that estimated with the annual data at island level (see Table 3). A similar situation was observed for Tenerife (61.96 vs. 32.38 DID). In contrast, the data obtained for both approaches were similar for Gran Canaria. In La Palma, the dispensation level was 15 points above the estimated value using the data at the island level but with wider 95% confidence intervals. In addition, the estimated variation rates were also different, 3.94% vs. 2.45% DID/year. The same conclusions can be drawn for the variation rates in the rest of the islands.

A possible explanation could be the type of data used for the estimation of the two parameters of interest, the dispensation level and the variation rate; specifically, the data on the variation of the DID over time for each of the municipalities that make up each island, whose heterogeneity is highly marked (Supplementary Table S1).

In Gran Canaria, for example, DID varied from 22.9DID in Artenara to 115DID in Moyá (see Supplementary Table S1), while in Lanzarote they ranged from 15.2DID in Teguise to 52.8DID in Arrecife. A similar pattern was observed in Fuerteventura, where the values ranged from 22.4 DID in La Oliva to 40.3 DID in Antigua.

In the case of Tenerife this is comparable, with values between 17.1 at El Tanque and 109.8 at Santa Cruz de Tenerife. In El Hierro, El Pinar stands out with a DID of 55.1. This is almost double that of the other two municipalities on the island. On the other hand, the greatest variations were observed in La Gomera, with a range from 31.8 DID in Valle Hermoso to 118.4 DID in Agulo, while in La Palma the greatest difference was found between two neighboring municipalities, Breña Alta with 151.7 DID compared to 35.0 DID in Breña Baja. Each municipality’s variability over the period should be added to this variability.

This variability implies a high uncertainty in the estimation of the parameters of interest. This is reflected in their respective 95% confidence intervals. The variance (s^2^) obtained using the municipal data was 40.3 (see Table 6). This is twenty times the value obtained using the annual data adjustment (s^2^ = 1.9), as shown in Table 3.

However, the results obtained in this work suggest that consumption may be higher in both the urban and rural areas at the island level, but the analysis at the municipal level seems to confirm also this result. A detailed analysis for each municipality shows the differences between them. Different types of municipalities can be distinguished. The more ruralized municipalities, with lower population density, ageing population, low socioeconomic status and limited access to the urban center, can show two types of prescription patterns: (1) municipalities with low DID values, such as Artenera and Tejeda in Gran Canaria; Vallehermoso and Hermigua in La Gomera; Tinajo and Teguise in Lanzarote, El Tanque and Arafo in Tenerife, and (2) municipalities with high DID values, such as Barlovento in La Palma; Ingenio in Gran Canaria; La Guancha in Tenerife and El Pinar in El Hierro.

The municipalities of Breña Baja in La Palma; San Miguel de Abona and El Rosario in Tenerife; and Santa Brígida in Gran Canaria also have low levels of DID. However, all these municipalities are considered as residential areas with easy access to the urban area, where many inhabitants live in the town but travel daily to another municipality for their work in the service and/or administration sector. Finally, the municipalities considered as tourist and urban areas present low DID values compared to the capital of the island, mainly urban area. This is the case of Adeje and Arona in Tenerife; San Bartolome de Tirajana, Mogán and Aguimes in Gran Canaria; Yaiza in Lanzarote and La Oliva in Fuerteventura.

### 4.4 Limitations and strengths

The results of the present study should be interpreted in the context of its limitations. The dataset only provides information on dispensed prescriptions. This may not reflect the actual use of ADs. Furthermore, the dataset included prescriptions for all residents of the Canary Islands who are registered in the provincial population register and have access to the Spanish national health service. This system guarantees a universal system of healthcare for all the inhabitants of Spain. Thus, the extrapolation of the results to other healthcare systems may be limited.

Secondly, the DID data at the island and provincial level are more accurate since they included all the population who live in that zone. At the municipal level, there is a bias, but it is difficult to quantify because the prescription is associated with the postal code (ZP) and not with the municipality in which the patients live. In such a situation, the small municipalities (<2,000 inhabitants) with a single community pharmacy have levels of variation in sales of around 10%–20%. On the other hand, in municipalities with two or more community pharmacies, the pattern was the same, although in some ZIPs, the level of variation was less than 10%, but in some this exceeded 20%, especially in the capital cities of Tenerife, Gran Canaria and Lanzarote.

However, several strengths include the analysis of consumption based on data on ADs sold in the pharmacies under prescription, and the length of the time period of the study. In addition, the use of the ATC/DDD methodology also adds strength to the results. This methodology allowed the aggregation of drug data independently of strength and dosage form and the comparison of drug use data in a standardized way across the selected settings. Although prescription AD use has been studied in various countries in the last 20 years, few studies in Europe have focused on local and isolated zones such as islands to establish a pattern of use of prescription ADs, which is a novel approach.

## 5 Conclusion

The results obtained in this study are in line with those reported in other European countries and Spanish regions, where a general pattern of increasing consumption has been observed over the past 2 decades. However, the relative levels of consumption between the islands show some notable differences. In particular, dispensation levels and variation rates were higher than the European countries average in the two main islands and La Palma, while the remaining islands had a lower than average but with a variation rate equal or higher than average one. Global trends in the use of three subgroups of ADs in developed countries were comparable to the results obtained in the Canary Islands. In this context, geographical location and cultural factors may partly explain the disparities in ADs consumption observed, whereas the urbanization grade and the population density seems to have no influence in global consumption of ADs and the various subgroups and ADs drugs at the island level. In this respect, all AD drugs are equally available regardless of the geographical area of residence or socio-economic status although the type of AD and ranking varied slightly between islands due to the differences in general medical practice.

At the municipal level, the heterogeneity of trends and patterns has made interpretation of the data very difficult. Factors analyzed individually do not explain the differences in ADs use between rural and urban municipalities. A combination of these factors could contribute to a case-by-case explanation.

In conclusion, this study provides a picture of the distribution of AD use in the population of the Canary Islands and, in particular, in the different islands where social and cultural traditions could be determinants to explain this pattern, through the simultaneous examination of several socioeconomic and demographic factors.

The findings provide relevant information regarding the use of ADs in the Canary Islands in relation to socioeconomic and demographic factors that should be taken into consideration when developing public health interventions.

## Data Availability

The original contributions presented in the study are included in the article/Supplementary Material, further inquiries can be directed to the corresponding author.
