# Supplementary material for: Antidepressant use in the Canary Islands (Spain): a retrospective study of provincial, island and municipal trends and associated factors
Source: Front Pharmacol. 2025 Jul 2;16:1511936. doi: 10.3389/fphar.2025.1511936 (PMC12263686; doi:10.3389/fphar.2025.1511936)
Supplement: Supplementary file 1 [file Table1.docx]

**Supplementary Material**

Table S1. Estimated marginal means for the different islands with their 95% confidence intervals. SE is the standard error for each island which depends on the number of municipalities on each island. Tenerife and Gran Canaria have thirty-one and twenty-eight municipalities, respectively. The island of El Hierro has only three municipalities. The remaining islands are divided into six municipalities, while La Palma is divided into 14.

| Island | Marginal mean | SE | df | 2.5% | 97.5% |
| --- | --- | --- | --- | --- | --- |
| Tenerife | 65.2 | 0.465 | 423 | 64.2 | 66.1 |
| El Hierro | 40.4 | 1.495 | 423 | 37.5 | 43.4 |
| Fuerteventura | 33.6 | 1.158 | 423 | 31.3 | 35.9 |
| Gran Canaria | 78.9 | 0.565 | 423 | 77.8 | 80.0 |
| La Gomera | 63.7 | 1.057 | 423 | 61.6 | 65.8 |
| La Palma | 88.6 | 0.718 | 423 | 87.2 | 90.0 |
| Lanzarote | 29.1 | 0.979 | 423 | 27.1 | 31.0 |

Table S1. Estimated marginal means for the different municipalities on each island with their 95% confidence intervals.

| Municipalities | Island | Marginal mean | 2.5% | 97.5% |
| --- | --- | --- | --- | --- |
| Adeje | Tenerife | 46.2 | 41.1 | 51.2 |
| Arafo | Tenerife | 33.1 | 28.0 | 38.2 |
| Arico | Tenerife | 52.3 | 47.2 | 57.4 |
| Arona | Tenerife | 56.9 | 51.8 | 62.0 |
| Buenavista del Norte | Tenerife | 86.5 | 81.4 | 91.6 |
| Candelaria | Tenerife | 83.1 | 78.0 | 88.2 |
| El Rosario | Tenerife | 37.2 | 32.1 | 42.3 |
| El Sauzal | Tenerife | 50.0 | 44.9 | 55.1 |
| El Tanque | Tenerife | 17.1 | 12.0 | 22.2 |
| Fasnia | Tenerife | 48.9 | 43.8 | 54.0 |
| Garachico | Tenerife | 91.1 | 86.0 | 96.2 |
| Granadilla de Abona | Tenerife | 53.4 | 48.4 | 58.5 |
| Guía de Isora | Tenerife | 57.2 | 52.1 | 62.2 |
| Güímar | Tenerife | 86.4 | 81.3 | 91.5 |
| Icod de los Vinos | Tenerife | 76.0 | 70.9 | 81.1 |
| La Guancha | Tenerife | 103.1 | 98.0 | 108.2 |
| La Matanza de Acentejo | Tenerife | 42.7 | 37.6 | 47.8 |
| La Orotava | Tenerife | 66.7 | 61.6 | 71.8 |
| La Victoria de Acentejo | Tenerife | 88.1 | 83.0 | 93.2 |
| Los Realejos | Tenerife | 79.7 | 74.6 | 84.8 |
| Los Silos | Tenerife | 60.4 | 55.3 | 65.5 |
| Puerto de la Cruz | Tenerife | 85.9 | 80.8 | 91.0 |
| San Cristóbal de La Laguna | Tenerife | 62.8 | 57.7 | 67.9 |
| San Juan de la Rambla | Tenerife | 69.8 | 64.7 | 74.9 |
| San Miguel de Abona | Tenerife | 19.5 | 14.4 | 24.6 |
| Santa Cruz de Tenerife | Tenerife | 109.8 | 104.7 | 114.9 |
| Santa Úrsula | Tenerife | 78.3 | 73.2 | 83.4 |
| Santiago del Teide | Tenerife | 45.5 | 40.4 | 50.6 |
| Tacoronte | Tenerife | 92.7 | 87.6 | 97.8 |
| Tegueste | Tenerife | 68.5 | 63.4 | 73.6 |
| Vilaflor de Chasna | Tenerife | 70.9 | 65.8 | 76.0 |
| El Pinar de El Hierro | El Hierro | 55.1 | 50.0 | 60.2 |
| Frontera | El Hierro | 32.0 | 26.9 | 37.1 |
| Valverde | El Hierro | 34.2 | 29.1 | 39.3 |
| Antigua | Fuerteventura | 40.3 | 35.2 | 45.4 |
| La Oliva | Fuerteventura | 22.4 | 17.3 | 27.5 |
| Pájara | Fuerteventura | 27.1 | 22.0 | 32.2 |
| Puerto del Rosario | Fuerteventura | 39.0 | 33.9 | 44.1 |
| Tuineje | Fuerteventura | 39.0 | 33.9 | 44.1 |
| Agaete | Gran Canaria | 79.7 | 74.6 | 84.8 |
| Agüímes | Gran Canaria | 69.6 | 64.5 | 74.6 |
| Artenara | Gran Canaria | 22.9 | 17.8 | 28.0 |
| Arucas | Gran Canaria | 88.6 | 83.5 | 93.7 |
| Firgas | Gran Canaria | 63.0 | 58.0 | 68.1 |
| Gáldar | Gran Canaria | 104.5 | 99.4 | 109.6 |
| Ingenio | Gran Canaria | 101.5 | 96.4 | 106.6 |
| La Aldea de San Nicolás | Gran Canaria | 74.4 | 69.4 | 79.5 |
| Las Palmas de Gran Canaria | Gran Canaria | 94.8 | 89.8 | 99.9 |
| Mogán | Gran Canaria | 53.7 | 48.6 | 58.7 |
| Moya | Gran Canaria | 115.0 | 109.9 | 120.1 |
| San Bartolomé de Tirajana | Gran Canaria | 67.4 | 62.3 | 72.5 |
| Santa Brígida | Gran Canaria | 65.3 | 60.2 | 70.4 |
| Santa Lucía de Tirajana | Gran Canaria | 83.2 | 78.2 | 88.3 |
| Santa María de Guía | Gran Canaria | 105.5 | 100.4 | 110.6 |
| Tejeda | Gran Canaria | 30.3 | 25.2 | 35.4 |
| Telde | Gran Canaria | 97.3 | 92.2 | 102.3 |
| Teror | Gran Canaria | 83.6 | 78.5 | 88.7 |
| Valleseco | Gran Canaria | 82.6 | 77.5 | 87.7 |
| Valsequillo | Gran Canaria | 84.5 | 79.4 | 89.6 |
| Vega de San Mateo | Gran Canaria | 88.9 | 83.8 | 94.0 |
| Agulo | La Gomera | 118.4 | 113.4 | 123.5 |
| Alajeró | La Gomera | 75.1 | 70.0 | 80.2 |
| Hermigua | La Gomera | 54.7 | 49.6 | 59.8 |
| San Sebastián de la Gomera | La Gomera | 58.3 | 53.2 | 63.4 |
| Valle Gran Rey | La Gomera | 43.8 | 38.7 | 48.8 |
| Vallehermoso | La Gomera | 31.8 | 26.7 | 36.9 |
| Barlovento | La Palma | 104.4 | 99.3 | 109.5 |
| Breña Alta | La Palma | 151.7 | 146.6 | 156.8 |
| Breña Baja | La Palma | 35.0 | 29.9 | 40.1 |
| El Paso | La Palma | 75.3 | 70.2 | 80.4 |
| Fuencaliente de la Palma | La Palma | 77.3 | 72.2 | 82.4 |
| Los Llanos de Aridane | La Palma | 110.5 | 105.4 | 115.6 |
| Puntagorda | La Palma | 81.3 | 76.2 | 86.3 |
| Puntallana | La Palma | 63.9 | 58.8 | 68.9 |
| San Andrés y Sauces | La Palma | 112.8 | 107.8 | 117.9 |
| Santa Cruz de la Palma | La Palma | 112.8 | 107.8 | 117.9 |
| Tazacorte | La Palma | 81.9 | 76.8 | 87.0 |
| Tijarafe | La Palma | 61.1 | 56.0 | 66.2 |
| Villa de Mazo | La Palma | 83.7 | 78.6 | 88.8 |
| Arrecife | Lanzarote | 52.8 | 47.7 | 57.9 |
| Haría | Lanzarote | 34.2 | 29.1 | 39.3 |
| San Bartolome | Lanzarote | 19.6 | 14.5 | 24.6 |
| Teguise | Lanzarote | 15.2 | 10.1 | 20.3 |
| Tías | Lanzarote | 40.3 | 35.2 | 45.4 |
| Tinajo | Lanzarote | 19.0 | 13.9 | 24.0 |
| Yaiza | Lanzarote | 22.3 | 17.2 | 27.4 |
